# Supplementary material for: Consistent administration of cetuximab is associated with favorable outcomes in recurrent/metastatic head and neck squamous cell carcinoma in an endemic carcinogen exposure area: a retrospective observational study
Source: PeerJ. 2020 Sep 10;8:e9862. doi: 10.7717/peerj.9862 (PMC7487150; doi:10.7717/peerj.9862)
Supplement: Supplemental Information 2 [file peerj-08-9862-s002.doc]

Supplementary Table S1. Baseline characteristics in the entire cohort according to the groups of cetuximab cycles.

| Variables | Cetuximab cycles | | *P* |
| --- | --- | --- | --- |
|  | < 11 cycles | ≥ 11 cycles |  |
| Cetuximab cycle (mean ± SD) | 7.6 ± 2.2 | 17.3 ± 3.7 | **<0.001** |
| Age, years (mean ± SD) | 54.7 ± 8.9 | 55.4 ± 10.7 | 0.666 |
| Alcohol | 33 (71.7%) | 38 (63.3%) | 0.361 |
| Betel nuts | 35 (76.1%) | 41 (68.3%) | 0.364 |
| Smoking | 34 (73.9%) | 45 (75.0%) | 0.651 |
| Primary sites |  |  | 0.422 |
| HPC | 9 (19.6%) | 12 (20.0%) |  |
| OC | 32 (69.6%) | 36 (60.0%) |  |
| OPC | 5 (10.9%) | 12 (20.0%) |  |
| Grade |  |  | 0.973 |
| 1 | 11 (23.9%) | 17 (28.3%) |  |
| 2 | 26 (56.5%) | 31 (51.7%) |  |
| 3 | 7 (15.2%) | 9 (15.0%) |  |
| Unknown | 2 (4.3%) | 3 (5.0%) |  |
| Margin positivity | 5 (10.9%) | 6 (10.0%) | 0.692 |
| LVI, positive | 2 (4.3%) | 2 (3.3%) | 0.896 |
| PNI, positive | 5 (10.9%) | 4 (6.7%) | 0.729 |
| ENE, positive | 2 (4.3%) | 3 (5.0%) | 0.627 |
| Tumor size |  |  | 0.128 |
| T0 | 2 (4.3%) | - |  |
| T1 | 4 (8.7%) | 10 (16.7%) |  |
| T2 | 13 (28.3%) | 11 (18.3%) |  |
| T3 | 9 (19.6%) | 7 (11.7%) |  |
| T4 | 18 (39.1%) | 32 (53.3%) |  |
| Lymph node status |  |  | 0.582 |
| N0 | 9 (19.6%) | 18 (30.0%) |  |
| N1 | 6 (13.0%) | 6 (10.0%) |  |
| N2 | 27 (58.7%) | 29 (48.3%) |  |
| N3 | 4 (8.7%) | 7 (11.7%) |  |
| Stage at initial diagnosis |  |  | 0.194 |
| I | 2 (4.3%) | 7 (11.7%) |  |
| II | 4 (8.7%) | 2 (3.3%) |  |
| III | 7 (15.2%) | 4 (6.7%) |  |
| IV | 33 (71.7%) | 47 (78.3%) |  |
