# Supplementary material for: Consistent administration of cetuximab is associated with favorable outcomes in recurrent/metastatic head and neck squamous cell carcinoma in an endemic carcinogen exposure area: a retrospective observational study
Source: PeerJ. 2020 Sep 10;8:e9862. doi: 10.7717/peerj.9862 (PMC7487150; doi:10.7717/peerj.9862)
Supplement: Supplemental Information 3 [file peerj-08-9862-s003.doc]

Supplementary Table S2. Treatment modality according to the groups of cetuximab cycles

| Variables | Cetuximab cycles | | *P* |
| --- | --- | --- | --- |
|  | < 11 cycles | ≥ 11 cycles |  |
| Previous treatment |  |  |  |
| Surgery | 38 (82.6%) | 45 (75.0%) | 0.346 |
| Chemotherapy | 39 (84.8%) | 47 (78.3%) | 0.400 |
| CRT | 40 (87.0%) | 45 (75.0%) | 0.126 |
| CRT-refractory | 19 (41.3%) | 15 (25.0%) | 0.075 |
| Cetuximab applied reason |  |  | 0.261 |
| Metastasis | 31 (67.4%) | 34 (56.7%) |  |
| Recurrence | 15 (32.6%) | 26 (43.3%) |  |
| Regimen of chemotherapy |  |  | 0.373 |
| PF | 36 (78.3%) | 40 (66.7%) |  |
| Taxane-based | 5 (10.9%) | 12 (20.0%) |  |
| Others | 5 (10.9%) | 8 (13.3%) |  |
| Platinum |  |  | 0.377 |
| Cisplatin | 39 (84.8%) | 46 (76.7%) |  |
| Carboplatin | 1 (2.2%) | 4 (6.7%) |  |
| Chemotherapy dose |  |  | 0.180 |
| 60/800 | 19 (41.3%) | 17 (28.3%) |  |
| 75/1000 | 22 (47.8%) | 35 (58.3%) |  |
| Disease progressed | 46 (100%) | 59 (98.3%) |  |
| Median PFS (months, 95% CI) | 3.00 (3.00-3.00) | 6.00 (7.00-9.00) | **<0.001** |
| All-cause mortality | 32 (69.6%) | 36 (60.0%) |  |
| Median OS (months, 95% CI) | 4.46 (3.85-9.38) | 12.43 (8.39-16.93) | **0.002** |

.
