# Supplementary material for: Consistent administration of cetuximab is associated with favorable outcomes in recurrent/metastatic head and neck squamous cell carcinoma in an endemic carcinogen exposure area: a retrospective observational study
Source: PeerJ. 2020 Sep 10;8:e9862. doi: 10.7717/peerj.9862 (PMC7487150; doi:10.7717/peerj.9862)
Supplement: Supplemental Information 4 — P-value estimated using Fisher’s exact or chi-squared test appropriately. [file peerj-08-9862-s004.doc]

Supplementary Table S3. CT response according different stages

|  | Stage I-III | Stage IV | *P* |
| --- | --- | --- | --- |
| Cases | 26 | 80 |  |
| Response |  |  | 0.692 |
| CR | 0(0) | 1 (1.3%) |  |
| PR | 6 (23.1%) | 23 (28.8%) |  |
| SD | 7 (26.9%) | 14 (17.5%) |  |
| PD | 13 (50.0%) | 42 (52.5%) |  |
| ORR | 6 (23.1%) | 24 (30.0%) | 0.314 |
| DCR | 13 (50.0%) | 38 (47.5%) | 0.927 |

P-value estimated using Fisher’s exact or chi-squared test appropriately.
