# Supplementary material for: Consistent administration of cetuximab is associated with favorable outcomes in recurrent/metastatic head and neck squamous cell carcinoma in an endemic carcinogen exposure area: a retrospective observational study
Source: PeerJ. 2020 Sep 10;8:e9862. doi: 10.7717/peerj.9862 (PMC7487150; doi:10.7717/peerj.9862)
Supplement: Supplemental Information 5 — NA, not available. [file peerj-08-9862-s005.docx]

Supplementary Table S4. The impact of multiple endemic habits on PFS and OS.

| Alcohol | Betel nuts | Smoking | PFS | |  | OS | |
| --- | --- | --- | --- | --- | --- | --- | --- |
|  |  |  | HR (95%CI) | *P* |  | HR (95%CI) | *P* |
| No | No | No | Reference |  |  | Reference |  |
| No | No | **Yes** | NA |  |  | NA |  |
| No | **Yes** | No | 0.97 (0.14-6.90) | 0.977 |  | 0.89 (0.18-4.41) | 0.885 |
| **Yes** | No | No | 1.40 (0.27-7.23) | 0.686 |  | 0.45 (0.09-2.21) | 0.324 |
| No | **Yes** | **Yes** | NA |  |  | 1.66 (0.17-16.12) | 0.661 |
| **Yes** | No | **Yes** | NA |  |  | NA |  |
| **Yes** | **Yes** | No | 1.73 (0.29-10.34) | 0.549 |  | 0.72 (0.15-3.59) | 0.691 |
| **Yes** | **Yes** | **Yes** | 2.42 (0.44-13.24) | 0.308 |  | 2.26 (0.56-9.05) | 0.251 |

NA: not available.
